# Optimizing Agronomic Management Practices for Enhanced Radiation Capture and Improved Radiation Use Efficiency in Winter Wheat

**DOI:** 10.3390/plants13152036

**Published:** 2024-07-24

**Authors:** Haicheng Xu, Mei Liu, Chuanxing Li, Yuhai Tang, Qiqin Xue, Wanli Xiao, Dongyao Gao, Dianliang Peng, Xinglong Dai

**Affiliations:** 1 Shandong Provincial University Laboratory for Protected Horticulture, Weifang University of Science and Technology, Weifang 262700, China; xuhaich@126.com (H.X.); sdaulm@126.com (M.L.); tangyh209@163.com (Y.T.); xueqiqin@163.com (Q.X.); xiaowanli1818@163.com (W.X.); dongyaogao@163.com (D.G.); 2 Shouguang Vegetable Industry Development Center, Weifang 262700, China; lichuanxing413@163.com; 3 State Key Laboratory of Crop Biology, Agronomy College, Shandong Agricultural University, Tai’an 271018, China

**Keywords:** winter wheat, agronomic practice, aboveground biomass, radiation capture, radiation use efficiency, grain yield

## Abstract

Increased aboveground biomass is contingent on enhanced photosynthetically active radiation intercepted by the canopy (IPAR), improved radiation use efficiency (RUE), or both. We investigated whether and how optimized agronomic management practices promote IPAR and RUE. Four integrated agronomic management treatments, i.e., local traditional practice (LP), improved local traditional practice (ILP), high-yield agronomic management (HY), and improved high-yield agronomic management (IHY), were compared over two wheat (*Triticum aestivum* L.) growing seasons. The average grain yield obtained with IHY was 96% relative to that of HY and was 7% and 23% higher than that with ILP and LP, respectively. Both HY and IHY consistently supported large values of the leaf area index and IPAR fraction, thereby increasing total IPAR. Treatment HY showed increased pre-anthesis RUE, manifested as a higher specific leaf nitrogen content and whole-plant N nutrition index at anthesis. The highest pre-anthesis aboveground biomass was obtained with HY due to the highest pre-anthesis IPAR and RUE. Along with a higher canopy apparent photosynthetic rate, IHY produced higher post-anthesis aboveground biomass due to its higher post-anthesis IPAR and RUE. Treatment IHY had a slightly lower total IPAR but a similar total RUE and harvest index, thus producing a slightly lower grain yield relative to HY. These results demonstrate that the optimized agronomic management practice used under IHY effectively enhances radiation capture and improves radiation utilization. Additionally, the net profit for IHY was higher than that for HY, ILP, and LP by 8%, 11%, and 88%, respectively. Considering the high grain yield, high RUE and high economic benefits, we recommend IHY as the agronomic management practice in the target region, although further study of improvements in pre-anthesis RUE is required.

## 1. Introduction

In the Huang-Huai-Hai Plain of China, the winter wheat (*Triticum aestivum* L.)–summer maize (*Zea mays* L.) rotation is the most popular cropping system. Over decades, local farmers have developed a fertilization strategy emphasizing nitrogen (N) fertilizers while neglecting phosphorous (P) and potassium (K) fertilizers, leading to a very small increasing trend in yield per unit area, low fertilizer utilization efficiency, and low production benefits. Moreover, the conventional planting patterns of these farmers, including low plant density [1], early harvesting of summer maize, and early sowing of wheat [2], result in the waste of heat and solar radiation [3]. In addition, high temperatures in autumn and winter, combined with early sowing, can easily increase the risk of early spring frost damage to winter wheat [4]. Therefore, we have proposed an integrated management strategy that includes delaying sowing dates, increasing plant densities, and reducing N fertilizer inputs; here, we verify whether this approach can synergistically enhance both the yield and resource utilization efficiency of winter wheat.

Wheat production hinges on a combination of agronomic practices. Grain yield and resource efficiency are related to tillage method [5], sowing date [6,7], plant density [8], and fertilization and irrigation management [9,10]. Although research has shown that agronomic practices have significantly affected wheat yields and resource efficiencies, few studies have explored wheat yield formation and resource utilization under the deployment of integrated agronomic management practices. Bearing this in mind, an integrated agronomic management strategy including different sowing dates, plant densities, and fertilization managements was proposed [11,12]; applying this strategy has resulted in improved crop yields and resource use efficiencies [13,14,15]. In recent studies, we have clarified the differences in the yield formation and N efficiency of wheat [16], as well as N balance and water productivity [17], using four integrated agronomic management practices. However, the impact of integrated management on radiation capture and radiation utilization remains little understood.

The grain yields of wheat depend on both the total aboveground biomass and the harvest index [18,19]. These factors vary due to genetic and agronomic factors [20,21]. It is difficult to increase the harvest index for modern wheat cultivars, despite the fact that this could markedly enhance grain yield [22,23,24]. Aboveground biomass plays a leading role in light energy utilization and is expressed as the product of the photosynthetically active radiation intercepted by the canopy (IPAR) and the radiation use efficiency (RUE) [25]. Differences in sowing dates, plant densities, and N fertilization management can directly affect the canopy leaf area index (LAI), the IPAR fraction, and the photosynthetic rate, producing differences in IPAR, RUE, or both, which ultimately influence the total aboveground biomass and grain yield [23].

In general, delayed sowing reduces aboveground N uptake, N accumulation [26], and biomass production [27,28]. An appropriate increase in plant density could significantly improve the tiller number [8], canopy LAI, and aboveground biomass [29,30]. Increased plant density has been reported to compensate for a reduction in aboveground biomass and grain yield in response to delayed sowing [28]. Recent studies have suggested that delayed sowing maintains similar aboveground biomass at anthesis and maturity as normal sowing [31], and can dramatically improve N utilization efficiency and assimilate partitioning without impacting wheat yield [6,7]. Generally, winter wheat reacts to an increase in the N application rate by increasing the canopy IPAR [32,33,34]. To maximize photosynthetic capacity and growth potential, crops undergo a change in N allocation. At the leaf level, crops possess the ability to modulate leaf area per unit N invested by modifying the specific leaf N content (SLN) [35,36]. At the crop level, the expression of the photosynthetic response is denoted as RUE and decreases when the canopy SLN drops beneath a critical threshold [37,38]. The N nutrition index (NNI), a key indicator, can be used to describe the canopy N nutritional status, in particular during the pre-anthesis stages [39]. Significant linear relationships have been found between LAI and SLN and NNI in the pre-anthesis period [40].

In the present study, we applied four integrated agronomic management treatments, investigating the tiller number, LAI, fraction of IPAR, leaf N, SLN, NNI, and harvest index. We assessed whether and how optimized agronomic management practices may enhance IPAR and improve RUE, evaluating their relative contributions to aboveground biomass. We believe that our results provide valuable insight for the further improvement of agronomic management strategies to improve wheat yield and resource efficiency in target regions.

## 2. Results

### 2.1. Tiller Number and Ear-Bearing Tiller Percentage

**Figure 1:**
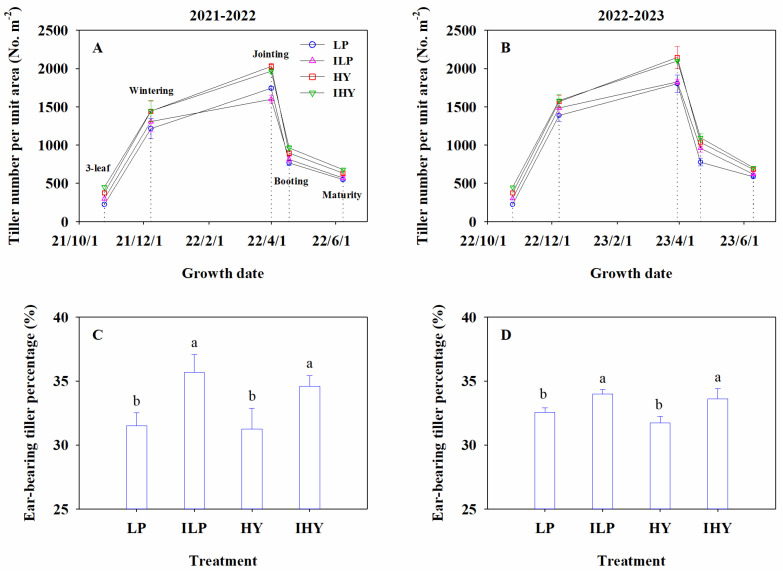
The dynamic of tiller number per unit area and ear-bearing tiller percentage under different integrated treatments in 2021–2022 and 2022–2023. (**A**,**B**), tiller number per unit area; (**C**,**D**), ear-bearing tiller percentage. LP, local traditional practice; ILP, improved local traditional practice; HY, high-yield agronomic management; IHY, improved high-yield agronomic management. Error bars represent the standard deviation of the mean. Different lowercase letters indicate significant differences (*p* < 0.05). Dotted lines are measurement periods.

The integrated management treatments significantly affected the tiller number per unit area and the ear-bearing tiller percentage. The tiller number increased with the time course of growth, reaching a peak value at jointing followed by a rapid decline (Figure 1A,B). Over the 2-year period, no marked difference in tiller number at the wintering stage was found in any integrated treatment. The tiller number at jointing differed remarkably across all integrated treatments, with a ranking of high-yield agronomic management (HY) ≈ improved high-yield agronomic management (IHY) > local traditional practice (LP) ≥ improved local traditional practice (ILP). The tiller number obtained with ILP was significantly lower than that with LP in 2021–2022, but no remarkable difference was observed between LP and ILP in 2022–2023. The tiller number at booting was the highest for HY and IHY, followed by ILP, and was lowest for LP. The number at maturity followed the ranking IHY > HY > ILP ≥ LP. With regard to ear-bearing tiller percentages, the rank order was ILP ≈ IHY > LP ≈ HY (Figure 1C,D). No significant differences were observed between LP and HY or between ILP and IHY. These results indicate that IHY achieved the highest spike number per unit area at maturity by producing the highest ear-bearing tiller percentage and highest tiller number at jointing.

### 2.2. LAI and Fraction of Intercepted PAR

**Figure 2:**
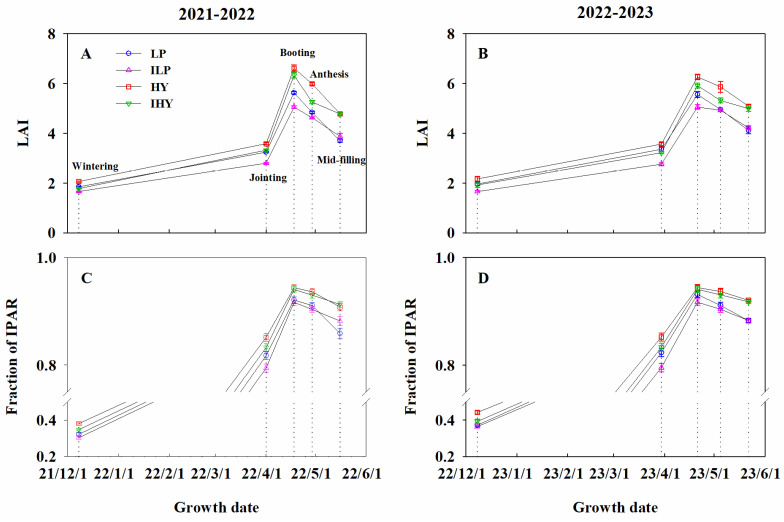
The dynamic of leaf area index (LAI) and fraction of IPAR under different integrated treatments in 2021–2022 and 2022–2023. (**A**,**B**), LAI; (**C**,**D**), fraction of IPAR. LP, local traditional practice; ILP, improved local traditional practice; HY, high-yield agronomic management; IHY, improved high-yield agronomic management. Error bars represent the standard deviation of the mean. Dotted lines are measurement periods.

Integrated management had a large effect on LAI. The LAI values increased with growth, peaked at booting, and gradually decreased thereafter (Figure 2A,B). Over the 2 years, HY consistently achieved the largest LAI throughout the growth period. The LAIs obtained with ILP in the pre-anthesis stages were the smallest among the integrated treatments. No significant differences were seen in LAI at the anthesis and mid-filling stages between ILP and LP. Compared to LP and ILP, IHY always produced a high LAI at the booting, anthesis, and mid-filling stages; however, it was notably lower than that with HY before anthesis and at anthesis.

The effects of management on IPAR fraction were significant. This fraction increased with growth, peaked at booting, and showed a gradual decline thereafter (Figure 2C,D). Throughout the 2 years, relative to LP and ILP, both HY and IHY consistently maintained a higher fraction. For IHY, it was slightly lower than that for HY before the jointing stage, while no marked differences were found between the two after booting. The fraction obtained with ILP was marginally lower than that obtained with LP before booting. Additionally, no marked difference in the fraction was observed between LP and ILP at the anthesis and mid-filling stages, except in 2021–2022, where the fraction at mid-filling for LP was slightly lower than that for ILP. These results show that both HY and IHY achieved a large fraction of IPAR by producing larger LAIs.

### 2.3. Leaf N, SLN, and NNI

**Figure 3:**
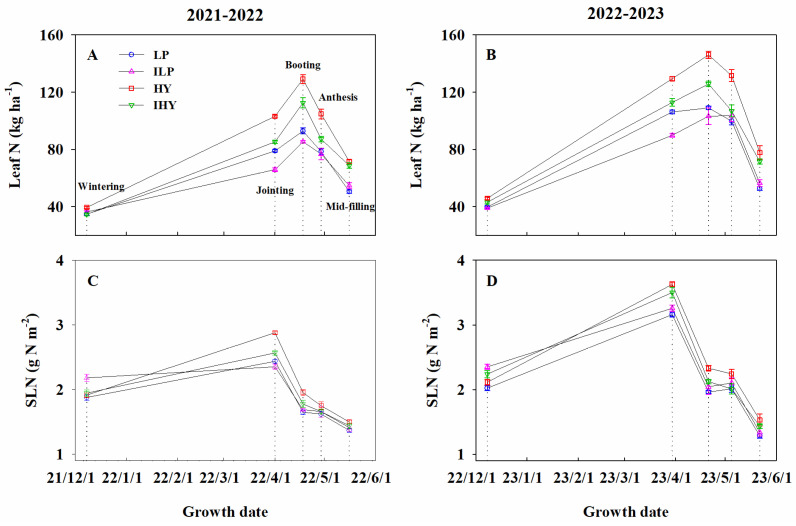
The dynamic of leaf N accumulation and specific leaf N content (SLN) under different integrated treatments in 2021–2022 and 2022–2023. (**A**,**B**), leaf N; (**C**,**D**), SLN. LP, local traditional practice; ILP, improved local traditional practice; HY, high-yield agronomic management; IHY, improved high-yield agronomic management. Error bars represent the standard deviation of the mean. Dotted lines are measurement periods.

The effects of integrated management treatments on leaf N accumulation and SLN were significant. As seen in the LAI trend, the value of leaf N increased with growth, peaked at booting, and showed a rapid decline thereafter (Figure 3A,B). Over the 2 years, leaf N was the highest for HY, followed by IHY and then LP (at the anthesis and mid-filling stages) and ILP (from wintering to booting). No significant differences in leaf N were observed between LP and ILP after anthesis, except in 2021–2022, where that obtained via LP at mid-filling was slightly lower than that obtained with ILP. The SLN reached its peak value at jointing and rapidly decreased thereafter (Figure 3C,D). Over the 2 years, the SLN at wintering for ILP was higher than that for other integrated treatments. After wintering, it was the highest for HY, followed by IHY and then LP. No remarkable differences in SLN at the anthesis and mid-filling stages were seen between IHY and ILP. Meanwhile, after the booting stage, no remarkable differences in SLN were found between ILP and LP. The average SLN for IHY was 95% of that for HY and 2% and 7% higher than that for ILP and LP, respectively.

**Figure 4:**
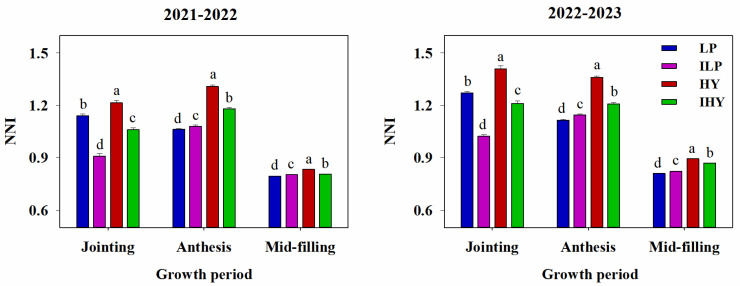
N nutrition index (NNI) under different integrated treatments in 2021–2022 and 2022–2023. LP, local traditional practice; ILP, improved local traditional practice; HY, high-yield agronomic management; IHY, improved high-yield agronomic management. Error bars represent the standard deviation of the mean. Different lowercase letters indicate significant differences (*p* < 0.05).

Integrated management treatments significantly affected the NNI (Figure 4). Over the 2 years, the values of NNI at jointing among all integrated treatments followed the ranking HY > LP > IHY > ILP. Except for ILP, the values for all integrated treatments were >1, implying luxury N consumption. At anthesis, whole-plant NNI values for all treatments were also >1 and followed the ranking HY > IHY > ILP > LP. At mid-filling, whole-spike NNI values for all treatments were <1 and followed a ranking of HY > IHY > ILP > LP, which was similar to the ranking observed for whole-plant NNI at anthesis.

### 2.4. Intercepted PAR and RUE

The management treatments significantly affected IPAR (Table 1). Over the 2 years, pre-anthesis IPAR among all treatments showed the following rank order: HY > IHY ≈ LP > ILP. The average pre-anthesis IPAR for IHY was 95% of that for HY and 4% higher than that for ILP. The post-anthesis IPAR was highest for IHY and HY, followed by ILP and then LP. The average post-anthesis IPAR for IHY was remarkably higher than that for ILP and LP, by 5% and 8%, respectively. For all treatments, the IPAR followed the ranking HY > IHY > LP > ILP. The average total IPAR for IHY was 97% of that for HY and 3% and 5% higher than that for LP and ILP, respectively.

**Figure 5:**
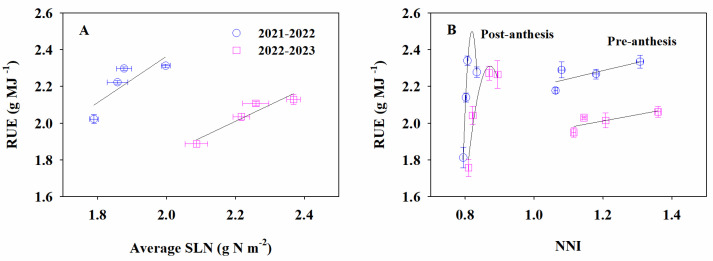
Radiation use efficiency (RUE) in relation to average specific leaf N content (SLN) and N nutrition index (NNI) in 2021–2022 and 2022–2023. (**A**), total RUE against average SLN; (**B**), pre-anthesis RUE against whole-plant NNI at anthesis and post-anthesis RUE against whole-spike NNI at mid-filling. LP, local traditional practice; ILP, improved local traditional practice; HY, high-yield agronomic management; IHY, improved high-yield agronomic management.

The effects of treatments on RUE were also significant (Table 1). Over the 2 years, pre-anthesis RUE followed the ranking HY > IHY ≈ ILP > LP. The average pre-anthesis RUE for IHY was 97% of that for HY and 4% higher than that for LP. For post-anthesis RUE, the order was IHY ≥ HY > ILP > LP. The average post-anthesis RUE for IHY was higher than that for HY, ILP, and LP by 2%, 10%, and 29%, respectively. The RUE over the whole period followed the ranking IHY ≈ HY > ILP > LP. The average total RUE for IHY was 3% and 13% higher than that for ILP and LP, respectively. Correlation analysis showed that total RUE was positively related to the average SLN (Figure 5A). Further, a linear relation was found between pre-anthesis RUE and whole-plant NNI at anthesis, while a curvilinear relationship was observed between post-anthesis RUE and whole-spike NNI in the mid-filling stage (Figure 5B).

### 2.5. Apparent Photosynthetic Rate of the Canopy

**Figure 6:**
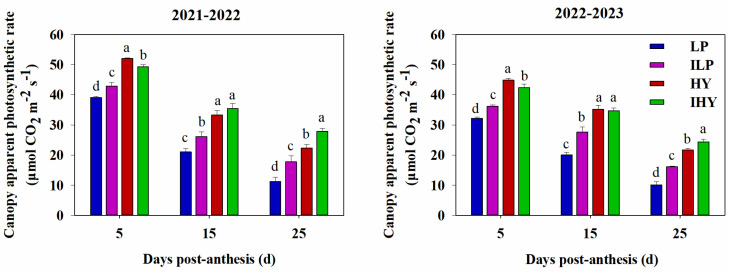
Canopy apparent photosynthetic rate under different integrated treatments in 2021–2022 and 2022–2023. LP, local traditional practice; ILP, improved local traditional practice; HY, high-yield agronomic management; IHY, improved high-yield agronomic management. Error bars represent the standard deviation of the mean. Different lowercase letters indicate significant differences (*p* < 0.05).

The treatments had a significant effect on the apparent photosynthetic rate in the canopy (Figure 6). The values decreased gradually during the grain-filling period. Over the 2 years, it was consistently the highest for HY or IHY, followed by ILP and then LP. The rate for IHY was slightly lower than that of HY in the early filling stage, roughly the same in the mid-filling stage, and markedly higher in the late-filling stage. These results indicate that the optimized agronomic practices used under IHY support high photosynthetic capacity during grain filling, promoting post-anthesis biomass production.

### 2.6. Aboveground Biomass, Harvest Index, and Grain Yield

The treatments significantly affected the aboveground biomass, harvest index, and grain yield (Table 2). Over the 2 years, the pre-anthesis aboveground biomass differed remarkably among all treatments, with a ranking of HY > IHY > ILP ≈ LP. The average value for IHY was 93% of that for HY and 4% higher than that for LP. For the post-anthesis aboveground biomass, the rank order was IHY ≥ HY > ILP > LP. The average value for IHY was 2%, 15%, and 39% higher than that for HY, ILP, and LP, respectively. The aboveground biomass at maturity followed a ranking of HY > IHY > ILP > LP. The average value for IHY was 96% of that for HY and was markedly higher than that for ILP and LP, by 8% and 16%, respectively. Over the 2 years, the lowest harvest index was obtained with LP. No significant differences in the harvest index were found among ILP, HY, and IHY. The grain yield followed the same ranking among all treatments as the total aboveground biomass at maturity. The average yield obtained with IHY was 96% of that with HY and was 7% and 23% higher than that with ILP and LP, respectively.

### 2.7. Relative Contributions

**Figure 7:**
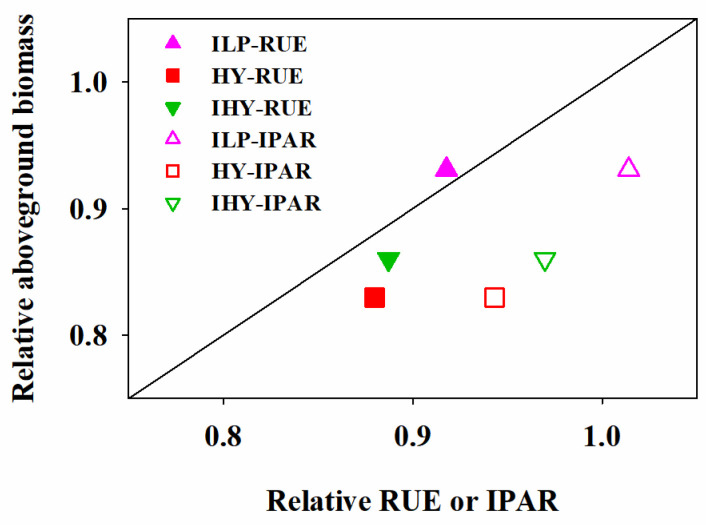
Relative aboveground biomass (LP cf. ILP, HY, and IHY) at maturity against either relative radiation use efficiency (RUE) or intercepted PAR (IPAR) across the two growing seasons. The line is y = x, which is provided for comparison. LP, local traditional practice; ILP, improved local traditional practice; HY, high-yield agronomic management; IHY, improved high-yield agronomic management.

We analyzed the aboveground biomass responses according to integrated agronomic management practices in terms of IPAR and RUE. The relative contributions of IPAR or RUE to the reduction in aboveground biomass obtained with LP differed significantly among the other three treatments (Figure 7). We compared the relative contributions of reduced IPAR or RUE for ILP, HY, and IHY relative to LP. The closer a data point is to the y = x line, the more important it was in determining the aboveground biomass loss. The reduction in RUE was a far more important determinant of the aboveground biomass than the reduction in IPAR. Decreases in either RUE or IPAR were a more important determinant of the aboveground biomass for ILP than for both HY and IHY. The relative contribution of RUE was slightly more important for IHY than for HY; however, no marked differences in the relative contribution of IPAR were found between IHY and HY.

### 2.8. Economic Benefits

Table 3 shows the differences in the input costs, outputs, and net profits between the four integrated management treatments. The input costs were the highest for HY, followed IHY, and then LP and ILP. The output among all treatments followed the same rank order as the grain yield due to the same unit wheat price. The net profit for IHY was higher than that for HY, ILP, and LP by 8%, 11%, and 88%, respectively.

## 3. Discussion

The HY treatment consistently achieved the highest total aboveground biomass and grain yield. The optimized agronomic management practice adopted under IHY also produced a high amount of aboveground biomass and a high harvest index, ultimately achieving a high level of grain yield. The lowest harvest index was obtained using LP, while no marked difference was seen among the other three integrated treatments. That is, the grain yields differed remarkably among practices, mainly due to the notable differences in the total aboveground biomass. Carbohydrates synthesized in the post-anthesis stage can contribute 60–90% of the final grain yield in wheat [41,42]. On average, in our study, the aboveground post-anthesis biomass contributed 75–84% of the grain yield. Over the 2 years, the highest post-anthesis aboveground biomass was obtained with IHY, which had the highest post-anthesis IPAR and RUE. The lowest biomass was obtained with LP due to its low IPAR and RUE values. Generally, IPAR yields decreased gradually with delayed sowing [43] but increased notably with increased N input [34] and plant density [30,44]. In the final analysis, enhanced radiation capture was achieved by improving the tiller number, canopy LAI, and the IPAR fraction. Taken together, our results demonstrate that increased plant density combined with optimized N management practices can compensate for reductions in canopy IPAR caused by delayed sowing.

RUE is calculated as the slope of the linear relationship between the aboveground biomass and IPAR [30]. Improvements in RUE are made possible by enhancing the photosynthetic capacity of the canopy. The photosynthetic capacity is commonly determined by the leaf N content. High levels of N accumulation enhance the photosynthetic capacity of the canopy and delay leaf senescence [7]. In this study, the leaf N was the highest for HY, followed by IHY and then LP and ILP, over the entire growth period. Along with high levels of leaf N and high LAI, both the HY and IHY treatments consistently maintained a higher apparent photosynthetic rate in the canopy during the grain filling period compared to LP and ILP. However, both LAI and leaf N obtained with HY decreased faster than that for IHY after anthesis. The photosynthetic capacity is closely related to SLN [35]. In our study, a strong linear relationship was observed between RUE and SLN over the whole growth period, in agreement with previous studies [38,45]. In addition, the pre-anthesis RUE was strongly synchronized with the average SLN in the pre-anthesis stage, following the same ranking of HY > IHY ≈ ILP > LP. The pre-anthesis RUE was slightly lower for IHY than for HY. Moreover, the average SLN in the post-anthesis stage exhibited the same trend as the average SLN in the pre-anthesis stage. However, the rank order for post-anthesis RUE was not exactly the same as that for the average SLN in the post-anthesis stage, which followed the ranking IHY ≥ HY > ILP > LP. Interestingly, despite the lower SLN post-anthesis, IHY achieved a similar or higher post-anthesis RUE than did HY. This may be attributable to its slower leaf senescence and better within-leaf N allocation post-anthesis. Further research is required to elucidate this.

The NNI is a widely used diagnostic tool for describing the N nutritional status of crops [46]. It is easily affected by soil tillage, the sowing date, and nutrient management [6,40,47]. Both the higher NNI at jointing and the lowest NNI at anthesis for LP were mainly due to the higher basal N input, earlier N topdressing date, and lower plant density. Similarly, the highest NNI was obtained with HY over the whole growth period, resulting from the high N input and optimized N topdressing date and ratio. The wheat grown under IHY obtained a lower NNI than that grown under HY due to the lower N input and higher plant density. Recent studies have suggested that the relationship between the NNI and RUE is linear from tillering to booting but becomes curvilinear at anthesis [40]. Considering the entire pre-anthesis period, the response of RUE to NNI is better described by a curvilinear or a segmented function [48]. In this study, a linear relation was found between the whole-plant NNI at anthesis and pre-anthesis RUE, whereas the response of the post-anthesis RUE to whole-spike NNI in the mid-filling stage was curvilinear, with a tendency for saturation at high NNI values. It is well established that spike N accumulation post-anthesis plays a crucial role in wheat yield. The whole-spike NNI is stable throughout the growth period [49,50]. However, an excessively high NNI may result in a small reduction in the RUE due to the greater energetic cost to build biomass [34,51]. Ultimately, despite a higher whole-spike NNI, the wheat grown under HY obtained a lower or similar post-anthesis RUE relative to IHY. In addition, whole-plant NNI values at anthesis were all > 1, implying that luxury N accumulated in whole plants. These results indicate that further improvements are possible in all integrated agronomic practices applied in our study, especially ILP and IHY.

Clearly, the lowest aboveground biomass and grain yield were obtained with LP, mainly due to poor agronomic management following the traditional planting practices of local farmers. By contrast, the highest total biomass and yield were obtained with HY due to the modified agronomic practices, regardless of the cost of resource inputs. In addition to N, P is also an essential crop nutrient which is critical for enhancing grain yield. Studies have shown that the total aboveground biomass and grain yield of wheat initially increased and then plateaued with the increase in P application [52]. Excessive P application did not enhance wheat yields but instead increased the negative environmental impacts through soil P leaching and runoff [53,54]. In this study, the wheat grown under HY obtained a higher P application compared to other treatments. However, IHY achieved 96% of the grain yield of HY under relatively low-input conditions. It appears that the integrated management practices used under HY produces surplus levels of soil P. Further study is still needed to assess the impacts of integrated management practices on P use efficiency and surpluses in future research.

Overall, the enhanced biomass for ILP, HY, and IHY is attributable to the increased IPAR or improved RUE relative to LP. However, the relative contributions of each to the reduction in aboveground biomass obtained with LP differed between integrated treatments. We found that decreases in the RUE were a far more important determinant of biomass than decreases in IPAR. Moreover, the relative contribution of the RUE was slightly more important for IHY than for HY and no notable difference was seen in the relative contribution of IPAR between IHY and HY. Indeed, the wheat grown under IHY achieved a slightly lower total IPAR but a similar total RUE as that grown under HY. These results imply that there may exist potential for the further enhancement of aboveground biomass production for IHY through the improvement or optimization of RUE.

## 4. Materials and Methods

### 4.1. Site Description

**Figure 8:**
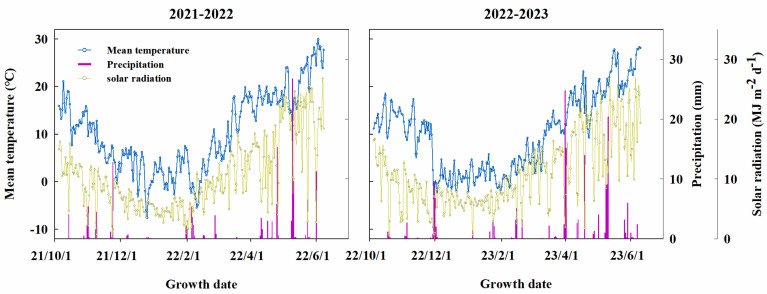
The mean temperature, precipitation, and solar radiation recorded during the winter wheat growing seasons in 2021–2022 and 2022–2023 in experimental fields.

Field experiments were conducted during the winter wheat growing season in 2021–2022 and 2022–2023 at the experimental station of Weifang University of Science and Technology in Weifang, Shandong Province, China (36°55′ N, 118°46′ E). The region has a temperate continental monsoon climate, with an annual average temperature, solar duration, total solar radiation, and total precipitation of 12.7 °C, 2548 h, 5190 MJ m^−2^, and 593.8 mm, respectively; the cumulative temperature above 10 °C is 4303 °C. The frost-free period over 1 year is 199 days. The soil is a sandy loam with 17.10 g kg^−1^ organic matter, 1.13 g kg^−1^ total N, 29.35 mg kg^−1^ available P, and 87.67 mg kg^−1^ available K in the upper 0.20 m of the soil before seeding in October 2021. Daily mean temperature, precipitation, and solar radiation during the two growing seasons are shown in Figure 8. All meteorological data used in the analysis were obtained from a local automatic weather station located <1000 m from the experimental field. The preceding crop grown in the experimental field was summer maize. Following harvest, all straw was plowed back into the soil every year.

### 4.2. Experimental Design

We used the widely planted winter wheat cultivar “Tainong 18” (with low tillering capacity and large ears) as the experimental material. Four integrated agronomic management treatments were conducted in a randomized complete block design with four replications: local traditional practice (LP), improved local traditional practice (ILP), high-yield agronomic management (HY), and improved high-yield agronomic management (IHY). The LP treatment followed the conventional planting pattern of local farmers. ILP treatment, based on LP, was designed to improve wheat yields and resource efficiency by delaying the sowing date, increasing the planting density, and decreasing fertilizer input. The HY treatment was designed to test the yield potential by adjusting agronomic management practices regardless of the resource input cost, mostly through increased plant density and fertilizer application rate, along with revised fertilization timing. The IHY treatment redesigned the local wheat production system, drawing on further delayed sowing dates, higher plant densities, and lower fertilizer application relative to HY treatment. The combination details of sowing date, plant density, and nutrients management used for each treatment are given in Table 4. Plant densities increased by 75 seeds m^−2^ with each integrated agronomic management treatment from LP (225 seeds m^−2^) to IHY (450 seeds m^−2^). Sowing dates for the treatments were 5, 10, 10, and 15 October, respectively. Urea (46% *w*/*w* N), calcium superphosphate (12% *w*/*w* P_2_O_5_), and potassium chloride (60% *w*/*w* K_2_O) were used as the N, P, and K fertilizers, respectively. Basal fertilizers were applied prior to planting, and additional fertilizers were applied at 5 cm soil depth in bands between rows. The basal and additional ratios of N fertilizer for the treatments were 6:4, 5:5, 4:6, and 4:6, respectively. N topdressing for LP was applied at regreening stage and that for ILP, HY, and IHY were applied at jointing stage. P fertilizers were applied as basal fertilizers for all treatments. K fertilizers for both LP and ILP were applied as basal fertilizers. The basal and additional ratios of K fertilizer for HY and IHY were 6:4. K topdressing for both HY and IHY were applied at jointing stage. Irrigation (75 mm each time) was performed after sowing, before wintering, at jointing, and at anthesis. Each experimental plot (3 × 10 m) contained 12 rows of wheat (positioned 0.25 m apart). Diseases and pests were controlled chemically every year. No significant incidence of diseases, weeds, or pests occurred in any experimental plot.

### 4.3. Sampling and Measurements

#### 4.3.1. Tiller Number and Ear-Bearing Tiller Percentage

Tiller number was counted at the three-leaf, wintering, jointing, booting, and maturity stages, respectively, in 1.5 m^2^ quadrats (six rows, 1 m apart) in each experimental plot, normalized according to the number per square meter. The ear-bearing tiller percentage was calculated by dividing the effective spike number by the maximum tiller number.

#### 4.3.2. LAI, Leaf N, SLN, and Aboveground Biomass

Plants from a 0.2 m^2^ quadrat (two rows, 0.4 m apart) were taken as samples for each plot at the wintering, jointing, booting, anthesis, mid-filling, and maturity stages. The plant samples were divided into three fractions (stems + sheaths, leaves, and spikes). The green area of the leaves was measured using a green area meter (Li-Cor 3100; Li-Cor, Lincoln, NE, USA). The dry matter of each plant component was weighed after oven drying to a constant mass at 75 °C for 48 h. Aboveground biomass (kg ha^−1^) was calculated as the summed dry weight of the measured plant components. N concentration was determined using the Kjeldahl method [55]. N accumulation in leaves (kg ha^−1^) was calculated by obtaining the dry mass and N concentration of the leaf. LAI, SLN (N m^−2^), and post-anthesis aboveground biomass (kg ha^−1^) was also calculated.

#### 4.3.3. NNI

The NNI was estimated in accordance with the ratio of actual N concentration (N_A_; %) and critical N concentration (N_C_; %). Based on the dilution curve for winter wheat described by Justes et al. [56], whole-plant N_C_ at pre-anthesis was calculated as follows:

N_C_ = 5.35 × W_P_^–0.44^ (1)

We focused on the N nutritional status of spikes instead of whole plants post-anthesis, following the dilution curve described by Zhao et al. [50]. Whole-spike N_C_ in the mid-filling stage was calculated as follows:

N_C_ = 2.85 × W_S_^−0.17^ (2)

where W_P_ (t ha^−1^) is the total aboveground dry weight per unit area pre-anthesis, and W_S_ is (t ha^−1^) the spike dry weight per unit area in the mid-filling stage.

#### 4.3.4. Intercepted PAR and RUE

At all stages, incident and transmitted photosynthetically active radiation (PAR) were measured on clear, cloudless days around noon (±1 h) utilizing a SunScan Canopy Analysis System (Delta-T, Cambridge, UK). The PAR above the canopy and at the ground level was measured at the same time using a portable probe placed at right angles to the rows and parallel to the surface at three locations in each plot. After anthesis, the transmitted PAR was determined as described by Sandaña et al. [57]. The fraction of IPAR was calculated as the ratio of the difference between incident and transmitted PAR to incident PAR. Stage IPAR (MJ m^−2^) was calculated as the product of the averaged fraction of IPAR of two consecutive sampling stages and the incident PAR between them [58]. The conversion factor for solar radiation to incident PAR was 0.5 [59]. RUE (g MJ^−1^) was calculated by dividing accumulated aboveground biomass by IPAR, pre- and post-anthesis and over the whole growth period.

#### 4.3.5. Canopy Apparent Photosynthetic Rate

Following the method described by Garrity et al. [60], the apparent photosynthetic rate in the canopy in the early filling stage (anthesis + 5 days), mid-filling stage (anthesis + 15 days), and late-filling stage (anthesis + 25 days) was measured using a portable infrared gas analysis system (GXH-3052L; Junfang Institute of Physics and Chemistry, Beijing, China). The assimilation chamber was constructed from polyester film (allowing >95% light transmittance) and was 0.70 m long × 0.60 m wide × 1.20 m high. Air in the assimilation chamber was mixed using a built-in fan 0.30 m in diameter. Changes in CO_2_ concentrations in the assimilation chamber were measured between 9:30 and 11:30 a.m. on clear, cloudless days. The assimilation chamber was opened before measurements to keep the CO_2_ concentration in the assimilation chamber equal to atmospheric CO_2_ concentrations. Two-row wheat plants were subsequently enclosed in the assimilation chamber and fully exposed to sunlight. The rapid decline in CO_2_ concentration was recorded until there was a steady decrease between 380 and 280 µmol mol^−1^ for at least 60 s [61]. The apparent photosynthetic rate of the canopy was calculated as described by Chen et al. [62].

#### 4.3.6. Grain Yield and Harvest Index

At maturity, all spikes from a 3.0 m^2^ quadrat (six rows, 2 m apart) in each experimental plot were collected and threshed. The grains were naturally air-dried, weighed, and adjusted to the standard 13% moisture content. Harvest index was defined as the ratio of grain yield to total aboveground biomass.

### 4.4. Statistical Analysis

The statistical analyses were performed with DPS ver. 7.05 software (Hangzhou Ruifeng Information Technology Co., Ltd., Hangzhou, China). Multiple comparisons were performed after a preliminary F-test. Differences between means were evaluated based on the least significant difference at *p* < 0.05. Figures were drawn using SigmaPlot 12.5 (Systat Software, San Jose, CA, USA).

## 5. Conclusions

This study investigated whether and how integrated agronomic management practices affect aboveground biomass production and grain yield in wheat. Both HY and IHY consistently supported a large LAI and fraction of IPAR values, thereby increasing the total IPAR. HY had increased pre-anthesis RUE with higher SLN and whole-plant NNI. The highest pre-anthesis aboveground biomass was obtained with HY, which had the highest pre-anthesis IPAR and RUE. Along with a high photosynthetic rate in the canopy, IHY produced higher aboveground biomass post-anthesis due to its higher post-anthesis IPAR and RUE. It also achieved a slightly lower total IPAR but a similar total RUE and harvest index, thus producing a slightly lower grain yield than HY. In conclusion, the optimized agronomic management practices (appropriately delayed sowing date, increased plant density, and optimized fertilization management) used under IHY effectively enhanced the radiation captured and improved radiation utilization. Considering the high grain yield, high RUE, and high economic benefits, IHY can be recommended as an optimal agronomic management practice in the target region, although further research on improvements in pre-anthesis RUE are required.

## Figures and Tables

**Table 1:** Intercepted PAR (IPAR) and radiation use efficiency (RUE) at pre-anthesis, post-anthesis, and whole growth stages under different integrated treatments in 2021–2022 and 2022–2023.

Year	Treatment ^(1)^	Pre-Anthesis		Post-Anthesis		Whole Growth Stage	
		IPAR (MJ m^−2^)	RUE (g MJ^−1^)	IPAR (MJ m^−2^)	RUE (g MJ^−1^)	IPAR (MJ m^−2^)	RUE (g MJ^−1^)
2021–2022	LP	446.06 b	2.18 c	330.01 c	1.81 d	776.07 c	2.02 c
	ILP	427.69 c	2.29 b	337.94 b	2.14 c	765.63 d	2.22 b
	HY	472.47 a	2.34 a	350.62 a	2.28 b	823.08 a	2.31 a
	IHY	451.23 b	2.27 b	353.28 a	2.34 a	804.51 b	2.30 a
2022–2023	LP	573.50 b	1.95 c	283.58 c	1.76 c	857.08 c	1.89 c
	ILP	552.83 c	2.03 b	292.22 b	2.04 b	845.05 d	2.03 b
	HY	603.21 a	2.06 a	304.97 a	2.27 a	908.18 a	2.13 a
	IHY	573.10 b	2.01 b	306.43 a	2.28 a	879.54 b	2.11 a
*p*-value							
Year (Y)		0.0001	0.0001	0.0001	0.0016	0.0001	0.0001
Treatment (T)		0.0001	0.0001	0.0001	0.0001	0.0001	0.0001
Y × T		0.5072	0.5076	0.9430	0.3422	0.0017	0.0146

^(1)^ LP, local traditional practice; ILP, improved local traditional practice; HY, high-yield agronomic management; IHY, improved high-yield agronomic management. Values followed by different letters within a column in the same year are significantly different at *p* < 0.05.

**Table 2:** Aboveground biomass at the pre-anthesis, post-anthesis, and maturity stages, harvest index, and grain yield under different integrated treatments in 2021–2022 and 2022–2023.

Year	Treatment ^(1)^	Aboveground Biomass (kg ha^−1^)			Harvest Index	Grain Yield (kg ha^−1^)
		Pre-Anthesis	Post-Anthesis	At Maturity		
2021–2022	LP	9705.18 c	5984.21 d	15,689.39 d	0.46 b	7295.24 d
	ILP	9785.65 c	7234.35 c	17,020.00 c	0.49 a	8357.35 c
	HY	11,033.17 a	7985.08 b	19,018.25 a	0.49 a	9409.29 a
	IHY	10,229.71 b	8262.07 a	18,491.78 b	0.49 a	9010.58 b
2022–2023	LP	11,169.64 c	4986.64 c	16,156.29 d	0.46 b	7418.22 d
	ILP	11,224.76 c	5964.68 b	17,189.43 c	0.49 a	8485.64 c
	HY	12,439.87 a	6908.11 a	19,347.98 a	0.49 a	9489.69 a
	IHY	11,544.28 b	6975.12 a	18,519.40 b	0.49 a	9086.09 b
*p*-value						
Year (Y)		0.0001	0.0001	0.0001	0.3070	0.0004
Treatment (T)		0.0001	0.0001	0.0001	0.0001	0.0001
Y × T		0.7745	0.1493	0.0320	0.1354	0.8159

^(1)^ LP, local traditional practice; ILP, improved local traditional practice; HY, high-yield agronomic management; IHY, improved high-yield agronomic management. Values followed by different letters within a column in the same year are significantly different at *p* < 0.05.

**Table 3:** The input cost, output, and net profit under different integrated treatments averaged across 2021–2023.

Treatment ^(1)^	Input (CNY)							Output (CNY)	Net Profit (CNY)
	Fertilizers	Seeds	Irrigation	Mechanical Operation	Pesticide	Labor	Total		
LP	3160.27	450.00	600.00	3300.00	450.00	6000.00	13,960.27	18,391.82	4431.55
ILP	2576.49	600.00	600.00	3300.00	450.00	6000.00	13,526.49	21,053.73	7527.24
HY	4808.97	750.00	600.00	3300.00	450.00	6000.00	15,908.97	23,623.72	7714.76
IHY	3027.80	900.00	600.00	3300.00	450.00	6000.00	14,277.80	22,620.85	8343.05

^(1)^ LP, local traditional practice; ILP, improved local traditional practice; HY, high-yield agronomic management; IHY, improved high-yield agronomic management.

**Table 4:** The sowing date, plant density, and nutrients management used in the different integrated treatments.

Treatment ^(1)^	Sowing Date (m/d)	Plant Density (Seeds m^−2^)	The Stage and Amount of Fertilizer Application (kg ha^−1^)			
			Fertilizer	Before Seeding	Regreening	Jointing
LP	10/5	225	N	189	126	–
			P_2_O_5_	120	–	–
			K_2_O	30	–	–
ILP	10/10	300	N	105	–	105
			P_2_O_5_	90	–	–
			K_2_O	75	–	–
HY	10/10	375	N	126	–	189
			P_2_O_5_	210	–	–
			K_2_O	90	–	60
IHY	10/15	450	N	96	–	144
			P_2_O_5_	120	–	–
			K_2_O	45	–	30

^(1)^ LP, local traditional practice; ILP, improved local traditional practice; HY, high-yield agronomic management; IHY, improved high-yield agronomic management. –, no data.

## Data Availability

The original contributions presented in the study are included in the article and further inquiries can be directed to the corresponding authors.

## References

[1] Zhang J., Wu T.H., Dai X.L., Wang X.Z., Li H.M., Jiang M.Y., He M.R. (2015). Effects of plant density and nitrogen level on nitrogen uptake and utilization of winter wheat. Acta Ecol. Sin..

[2] Zhao H.L., Shar A.G., Li S., Chen Y.L., Shi J.L., Zhang X.Y., Tian X.H. (2018). Effect of straw return mode on soil aggregation and aggregate carbon content in an annual maize-wheat double cropping system. Soil Till. Res..

[3] Liu Z., Gao J., Gao F., Dong S.T., Liu P., Zhao B., Zhang J.W. (2018). Integrated agronomic practices management improve yield and nitrogen balance in double cropping of winter wheat-summer maize. Field Crops Res..

[4] Wang J., Wang E.L., Yang X.G., Zhang F.S., Yin H. (2012). Increased yield potential of wheat-maize cropping system in the North China Plain by climate change adaptation. Clim. Change.

[5] Guan D.H., Zhang Y.S., Al-Kaisi M.M., Wang Q.Y., Zhang M.C., Li Z.H. (2015). Tillage practices effect on root distribution and water use efficiency of winter wheat under rain-fed condition in the north China plain. Soil Tillage Res..

[6] Yin L.J., Dai X.L., He M.R. (2018). Delayed sowing improves nitrogen utilization efficiency in winter wheat without impacting yield. Field Crops Res..

[7] Yin L.J., Xu H.C., Dong S.X., Chu J.P., Dai X.L., He M.R. (2019). Optimised nitrogen allocation favours improvement in canopy photosynthetic nitrogen-use efficiency: Evidence from late-sown winter wheat. Environ. Exp. Bot..

[8] Dai X.L., Zhou X.H., Jia D.Y., Xiao L.L., Kong H.B., He M.R. (2013). Managing the seeding rate to improve nitrogen-use efficiency of winter wheat. Field Crops Res..

[9] Lu J.S., Xiang Y.Z., Fan J.L., Zhang F.C., Hu T.T. (2021). Sustainable high grain yield, nitrogen use efficiency and water productivity can be achieved in wheatmaize rotation system by changing irrigation and fertilization strategy. Agric. Water Manag..

[10] Ye T.Y., Ma J.F., Zhang P., Shan S., Liu L.L., Tang L., Cao W.X., Liu B., Zhu Y. (2022). Interaction effects of irrigation and nitrogen on the coordination between crop water productivity and nitrogen use efficiency in wheat production on the north China plain. Agric. Water Manag..

[11] Chen X.P., Cui Z.L., Fan M.S., Vitousek P., Zhao M., Ma W.Q., Wang Z.L., Zhang W.J., Yan X.Y., Yang J.C. (2014). Producing more grain with lower environmental costs. Nature.

[12] Cui Z.L., Zhang H.Y., Chen X.P., Zhang C.C., Ma W.Q., Huang C.D., Zhang W.F., Mi G.H., Miao Y.X., Li X.L. (2018). Pursuing sustainable productivity with millions of smallholder farmers. Nature.

[13] Jin L.B., Cui H.Y., Li B., Zhang J.W., Dong S.T., Liu P. (2012). Effects of integrated agronomic management practices on yield and nitrogen efficiency of summer maize in North China. Field Crops Res..

[14] Zhang H., Hou D.P., Peng X.L., Ma B.J., Shao S.M., Jing W.J., Gu J.F., Liu L.J., Wang Z.Q., Liu Y.Y. (2019). Optimizing integrative cultivation management improves grain quality while increasing yield and nitrogen use efficiency in rice. J. Integr. Agric..

[15] Cheng Y., Dai X.L., Ren H., Wang Y.C., Liu P., He M.R. (2020). Precision double cropping synergistically improves wheat and maize yields as well as resource efficiency. Agron. J..

[16] Xu H.C., Dai X.L., Chu J.P., Wang Y.C., Yin L.J., Ma X., Dong S.X., He M.R. (2018). Integrated management strategy for improving the grain yield and nitrogen-use efficiency of winter wheat. J. Integr. Agric..

[17] Xu H.C., Liu M., Tang Y.H., Zhao F., Cao W.C., He M.R., Peng D.L., Dai X.L. (2023). Optimized management strategy increased grain yield, promoted nitrogen balance, and improved water productivity in winter wheat. Front. Plant Sci..

[18] Donald C.M., Hamblin J. (1976). The biological yield and harvest index of cereals as agronomic and plant breeding criteria. Adv. Agron..

[19] Rivera-Amado C., Trujillo-Negrellos E., Molero G., Reynolds M.P., Sylvester-Bradley R., Foulkes M.J. (2019). Optimizing dry-matter partitioning for increased spike growth, grain number and harvest index in spring wheat. Field Crops Res..

[20] Dai J., Bean B., Brown B., Bruening W., Edwards J., Flowers M., Karow R., Lee C., Morgan G., Ottman M. (2016). Harvest index and straw yield of five classes of wheat. Biomass Bioenergy.

[21] Porker K., Straight M., Hunt J.R. (2020). Evaluation of G×E×M interactions to increase harvest index and yield of early sown wheat. Front. Plant Sci..

[22] Fischer R.A. (2011). Wheat physiology: A review of recent developments. Crop Pasture Sci..

[23] Reynolds M., Foulkes J., Furbank R., Griffiths S., King J., Murchie E., Parry M., Slafer G. (2012). Achieving yield gains in wheat. Plant Cell Environ..

[24] Aisawi K.A.B., Reynolds M.P., Singh R.P., Foulkes M.J. (2015). The physiological basis of the genetic progress in yield potential of CIMMYT spring wheat cultivars from 1966 to 2009. Crop Sci..

[25] Zhang Z., Zhou X.B., Chen Y.H. (2016). Effects of irrigation and precision planting patterns on photosynthetic product of wheat. Agron. J..

[26] Ehdaie B., Waines J.G. (2001). Sowing date and nitrogen rate effects on dry matter and nitrogen partitioning in bread and durum wheat. Field Crops Res..

[27] Ferrise R., Triossi A., Stratonovitch P., Bindi M., Martre P. (2010). Sowing date and nitrogen fertilisation effects on dry matter and nitrogen dynamics for durum wheat: An experimental and simulation study. Field Crops Res..

[28] Shah F., Coulter J.A., Ye C., Wu W. (2020). Yield penalty due to delayed sowing of winter wheat and the mitigatory role of increased seeding rate. Eur. J. Agron..

[29] Bavec M., Vuković K., Mlakar S.G., Rozman Č., Bavec F. (2007). Leaf area index in winter wheat: Response on seed rate and nitrogen application by different varieties. J. Cent. Eur. Agric..

[30] Tao Z.Q., Wang D.M., Ma S.K., Yang S.S., Zhao G.C., Chang X.H. (2018). Light interception and radiation use efficiency response to tridimensional uniform sowing in winter wheat. J. Integr. Agric..

[31] Dai X.L., Wang Y.C., Dong X.C., Qian T.F., Yin L.J., Dong S.X., Chu J.P., He M.R. (2017). Delayed sowing can increase lodging resistance while maintaining grain yield and nitrogen use efficiency in winter wheat. Crop J..

[32] Gastal F., Nelson C.J. (1994). Nitrogen use within the growing leaf blade of tall fescue. Plant Physiol..

[33] Colnenne C., Meynard J.M., Roche R., Reau R. (2002). Effects of nitrogen deficiencies on autumnal growth of oilseed rape. Eur. J. Agron..

[34] Olesen J.E., Jorgensen L.N., Mortensen J.V. (2000). Irrigation strategy, nitrogen application and fungicide control in winter wheat on a sandy soil. II. Radiation interception and conversion. J. Agric. Sci..

[35] Sinclair T.R., Horie T. (1989). Leaf nitrogen, photosynthesis, and crop radiation use efficiency: A review. Crop Sci..

[36] Grindlay D.J.C. (1997). Towards an explanation of crop nitrogen demand based on the optimization of leaf nitrogen per unit leaf area. J. Agric. Sci..

[37] Hall A.J., Connor D.J., Sadras V.O. (1995). Radiation-use efficiency of sunflower crops: Effects of specific leaf nitrogen and ontogeny. Field Crops Res..

[38] Massignam A.M., Chapman S.C., Hammer G.L., Fukai S. (2009). Physiological determinants of maize and sunflower grain yield as affected by nitrogen supply. Field Crops Res..

[39] Ravier C., Meynard J.M., Cohan J.P., Gate P., Jeuffroy M.H. (2017). Early nitrogen deficiencies favor high yield, grain protein content and N use efficiency in wheat. Eur. J. Agron..

[40] Giunta F., Motzo R., Nemeh A., Bassu S. (2023). Changes in radiation capture and use in response to the nitrogen status of durum wheat cultivars at different developmental stages. Field Crops Res..

[41] Masoni A., Ercoli L., Mariotti M., Arduini I. (2007). Post-anthesis accumulation and remobilization of dry matter, nitrogen and phosphorus in durum wheat as affected by soil type. Eur. J. Agron..

[42] Dordas C. (2012). Variation in dry matter and nitrogen accumulation and remobilization in barley as affected by fertilization, cultivar, and source-sink relations. Eur. J. Agron..

[43] Zhang Z.Z., Cheng S., Fan P., Zhou N.B., Xing Z.P., Hu Y.J., Xu F.F., Guo B.W., Wei H.Y., Zhang H.C. (2023). Effects of sowing date and ecological points on yield and the temperature and radiation resources of semi-winter wheat. J. Integr. Agric..

[44] Zhang X.Q., Du S.Z., Xu Y.J., Cao C.F., Chen H. (2021). Reducing N application by increasing plant density based on evaluation of root, photosynthesis, N accumulation and yield of wheat. Agronomy.

[45] Fletcher A.L., Johnstone P.R., Chakwizira E., Brown H.E. (2013). Radiation capture and radiation use efficiency in response to N supply for crop species with contrasting canopies. Field Crops Res..

[46] Lemaire G., Jeuffroy M.H., Gastal F. (2008). Diagnosis tool for plant and crop N status in vegetative stage: Theory and practices for crop N management. Eur. J. Agron..

[47] Lemaire G., Gastal F. (1997). N uptake and distribution in plant canopies. Diagnosis of the Nitrogen Status in Crops.

[48] Olesen J.E., Berntsen J., Hansen E.M., Petersen B.M., Petersen J. (2002). Crop nitrogen demand and canopy area expansion in winter wheat during vegetative growth. Eur. J. Agron..

[49] Barraclough P.B., Lopez-Bellido R., Hawkesford M.J. (2014). Genotypic variation in the uptake, partitioning and remobilisation of nitrogen during grain-filling in wheat. Field Crops Res..

[50] Zhao B., Niu X.L., Ata-Ul-Karim S.T., Wang L.G., Duan A.W., Liu Z.D., Lemaire G. (2020). Determination of the post-anthesis nitrogen status using ear critical nitrogen dilution curve and its implications for nitrogen management in maize and wheat. Eur. J. Agron..

[51] Giunta F., Motzo R., Nemeh A., Pruneddu G. (2022). Durum wheat cultivars grown in Mediterranean environments can combine high grain nitrogen content with high grain yield. Eur. J. Agron..

[52] Li S.J., Chen X.X., Wang Z.K., Wu D.X., Wang M., Müeller T., Zou C.Q., Chen X.P., Zhang W. (2024). Phosphorus fertilizer management for high yields in intensive winter wheat-summer maize rotation system: Integrating phosphorus budget and soil available phosphorus. Field Crops Res..

[53] Yan X.J., Chen X.H., Tou C.Y., Luo Z.W., Ma C.C., Huang W.Q., Cui Z.L., Chen X.P., Wu L.Q., Zhang F.S. (2024). Exploring phosphorus fertiliser management in wheat production. Eur. J. Agron..

[54] Blackwell M., Darch T., Haslam R. (2019). Phosphorus use efficiency and fertilizers: Future opportunities for improvements. Front. Agric. Sci. Eng..

[55] Bremner J.M., Tabatabai M.A. (1972). Use of an ammonia electrode for determination of ammonium in kjeldahl analysis of soils. Commun. Soil Sci. Plant Anal..

[56] Justes E., Mary B., Meynard J.M., Machet J.M., Huché-Thélier L. (1994). Determination of a critical nitrogen dilution curve for winter wheat crops. Ann. Bot..

[57] Sandaña P., Ramírez M., Pinochet D. (2012). Radiation interception and radiation use efficiency of wheat and pea under different P availabilities. Field Crops Res..

[58] Acreche M.M., Briceño-Félix G., Martín Sánchez J.A., Slafer G.A. (2009). Radiation interception and use efficiency as affected by breeding in Mediterranean wheat. Field Crops Res..

[59] Sinclair T.R., Muchow R.C. (1999). Radiation use efficiency. Adv. Agron..

[60] Garrity D.P., Sullivan C.Y., Watts D.G. (1984). Rapidly determining sorghum canopy photosynthetic rates with a mobile field chamber. Agron. J..

[61] Zhang X., Hua Y.F., Liu Y.J., He M.R., Ju Z.C., Dai X.L. (2022). Wide belt sowing improves the grain yield of bread wheat by maintaining grain weight at the backdrop of increases in spike number. Front. Plant Sci..

[62] Chen M.Z., Zhang Y.L., Liang F.B., Tang J.Y., Ma P.C., Tian J.S., Jiang C.D., Zhang W.F. (2021). The net photosynthetic rate of the cotton boll-leaf system determines boll weight under various plant densities. Eur. J. Agron..

